# Gene expression profiles in asbestos-exposed epithelial and mesothelial lung cell lines

**DOI:** 10.1186/1471-2164-8-62

**Published:** 2007-03-01

**Authors:** Penny Nymark, Pamela M Lindholm, Mikko V Korpela, Leo Lahti, Salla Ruosaari, Samuel Kaski, Jaakko Hollmén, Sisko Anttila, Vuokko L Kinnula, Sakari Knuutila

**Affiliations:** 1 Health and Work Ability, Biological Mechanisms and Prevention of Work-related Diseases, Finnish Institute of Occupational Health, Helsinki, Finland; 2 Department of Pathology, Haartman Institute and HUSLAB, University of Helsinki and Helsinki University Central Hospital, Helsinki, Finland; 3 Helsinki Institute for Information Technology, Laboratory of Computer and Information Science, Helsinki University of Technology, Espoo, Finland; 4 Department of Medicine, Pulmonary Division, University of Helsinki and Helsinki University Hospital, Helsinki, Finland

## Abstract

**Background:**

Asbestos has been shown to cause chromosomal damage and DNA aberrations. Exposure to asbestos causes many lung diseases e.g. asbestosis, malignant mesothelioma, and lung cancer, but the disease-related processes are still largely unknown. We exposed the human cell lines A549, Beas-2B and Met5A to crocidolite asbestos and determined time-dependent gene expression profiles by using Affymetrix arrays. The hybridization data was analyzed by using an algorithm specifically designed for clustering of short time series expression data. A canonical correlation analysis was applied to identify correlations between the cell lines, and a Gene Ontology analysis method for the identification of enriched, differentially expressed biological processes.

**Results:**

We recognized a large number of previously known as well as new potential asbestos-associated genes and biological processes, and identified chromosomal regions enriched with genes potentially contributing to common responses to asbestos in these cell lines. These include genes such as the thioredoxin domain containing gene (*TXNDC*) and the potential tumor suppressor, BCL2/adenovirus E1B 19kD-interacting protein gene (*BNIP3L*), GO-terms such as "positive regulation of I-kappaB kinase/NF-kappaB cascade" and "positive regulation of transcription, DNA-dependent", and chromosomal regions such as 2p22, 9p13, and 14q21. We present the complete data sets as Additional files.

**Conclusion:**

This study identifies several interesting targets for further investigation in relation to asbestos-associated diseases.

## Background

Asbestos causes DNA double strand breaks [1], chromosomal aberrations, and abnormal chromosome segregation [2]. Asbestos fibre-induced genotoxicity has been proposed to be caused by both a direct interaction with the genetic material and also indirect effects via production of reactive oxygen species (ROS) [3]. The respiratory diseases linked to asbestos exposure include fibrotic lung disease, mesothelioma, and lung cancer. The pathogenesis and histopathology of asbestosis resemble that of idiopathic pulmonary fibrosis. Some specific genes contributing to the development of asbestosis and asbestos-related cancer have been described, as reviewed in [4]. However, the exact molecular mechanisms behind asbestos-associated carcinogenesis and fibrosis/asbestosis are thought to be very complex and involve several parallel pathways [4,5] that remain to be clarified.

Asbestos exposure has been reported to alter the expression of several genes involved in integrin-mediated signaling pathways, MAPK pathways, and NFKB/IKB pathways [6]. A recent study in a model for asbestos-induced oncogenesis demonstrated that the tumor necrosis factor, TNF-α, triggered by macrophages, induced activation of the NFKB cascade and thereby blocked apoptosis [7].

Studies from this laboratory as well as from others, have examined the gene copy number and expression changes in lung cancer patients with asbestos exposure and in mesothelioma. These have revealed a very complex pattern of chromosomal aberrations and altered gene expression, i.e. evidence for specific asbestos-associated aberrations and altered expression profile in these cancer genomes [8-10].

Given the evidence of association between fibrosis and cancer, it is clearly important to identify the specific genes involved in the inflammatory, fibrotic, and carcinogenetic processes in the lung following asbestos exposure, as reviewed in [4]. Chromosomal aberrations, together with more accurate information of gene expression alterations caused by asbestos, could provide valuable clues in the search for novel therapeutic targets.

We used three human lung cell lines to map the gene expression following crocidolite exposure. In addition to epithelial cells, mesothelial cells with features of both mesenchymal and epithelial cells [11], were used in the identification of asbestos-affected genetic pathways. Crocidolite was selected as it is probably the most pathogenic amphibole fibre, possibly due to its high iron content [4]. We report for the first time specific time-dependent genome-wide expression profiles in asbestos exposed human lung adenocarcinoma cells (A549), non-tumorigenic SV40-immortalized bronchial (Beas-2B), and pleural mesothelial cells (MeT5A).

## Results

**Figure 1:**
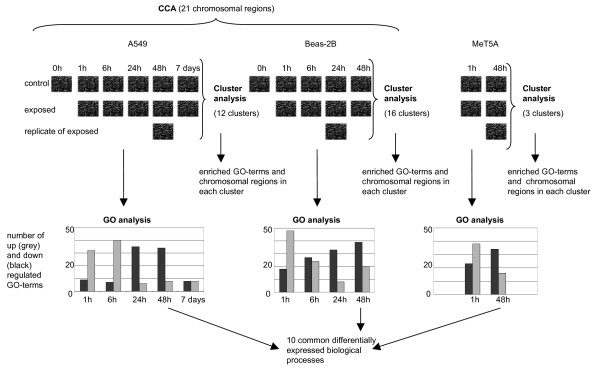
Overall study design.

The overall study design is illustrated in Fig. 1.

### Gene Ontology Analysis Results

An analysis to detect enriched Gene Ontology (GO) terms was carried out separately for all cell lines at several time points. We searched for branches with at least three enriched GO terms (p < 0.01) and the most detailed term containing less than 100 genes was listed (Additional file 1, GOanalysis). A total of 351 unique branches of the GO tree were enriched in at least one cell line at any one time point.

The GO terms at the 1 h and 48 h time points were compared between the cell lines to identify commonly enriched biological processes. Again, we restricted our focus to those branches of the GO tree that contained at least three enriched GO terms. No common processes were observed after 1 h exposure, whereas 10 common GO terms were identified after 48 h. We consider these biological processes to be potentially asbestos-associated since they were enriched in all cell lines. The number of genes belonging to the most detailed process of the branch ranged from 1 to 85 (Table 1). The complete results from all cell lines and all time points are appended in Additional file 1: GOanalysis.

### Cluster Analysis Results

**Figure 2:**
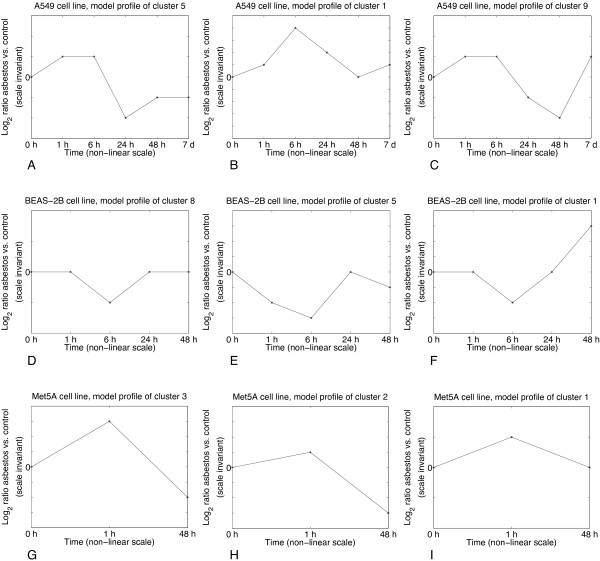
Profiles of the most significant clusters in A549 (A-C), Beas-2B (D-F) and MeT5A (G-I) cells. The X-axis shows the time points from 0 h-48 h (or in the case of A549, 7 days) and the Y-axis the expression ratio profile that is representative of all probe sets in the cluster. Due to the correlation-based distance measure used in the clustering method [48], the scale of the Y-axis is not relevant.

**Figure 3:**
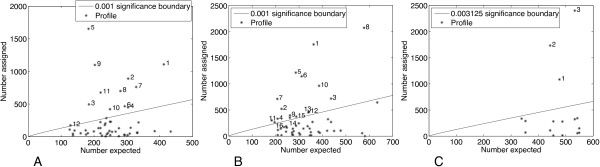
The significance of each cluster in A549 (A), Beas-2B (B), and MeT5A (C). The X-axis shows the number of expected probe sets and the Y-axis the number of assigned probe sets in each cluster. The clusters above the line are considered statistically significant based on permutation tests.

The differential expression according to asbestos-exposure at different time points was studied by cluster analysis and revealed 12 significant clusters in A549, 16 in Beas-2B, and 3 in MeT5A (Fig 2 and Additional files 2 and 3: A549clusters and Beas2Bclusters), with the total number of genes included in the cluster analysis results being 18,535. Fig. 3 illustrates the statistical significance of the attained clusters in each cell line. The clusters can be ordered with respect to their significance, based on the expected and realized number of probe sets assigned to the clusters. The clusters are not numbered in the exact order of significance due to the limits of numerical precision in the computation of p-values. The number of probes for each cluster ranged between 174 and 1,653, 266 and 2,073, and 1,085 and 2,403 (Additional file 4: Cluster_analysis), and the number of significant GO terms for each cluster ranged from 4 to 56, 1 to 45, and 10 to 52, whereas the number of enriched chromosomal regions ranged from 5 to 23, 2 to 15, and 8 to 11 (data not shown, available upon request) in the A549, Beas-2B and MeT5A cell lines, respectively. Further interpretation and discussion of the results focuses on the three most significant clusters in each cell line. These clusters were chosen according to the expected and realized number of probe sets assigned to them; in order of significance the clusters were 5, 9, and 1 in A549; 8, 1, and 5 in Beas-2B; and 1–3 in MeT5A (Fig. 2).

We compared the enriched biological processes in the clusters and in the GO analysis and found 29 common processes (data obtained upon request), of which 9 were significant in at least two clusters and at least two time points of the GO analysis (Table 2). The GO terms "negative regulation of survival gene product activity" and "positive regulation of transcription, DNA-dependent" matched with those obtained in the GO analysis of the 48 h time point and "cytoplasmic sequestering of NF-kappaB" was closely related to a third GO term in the 48 h analysis (Table 1).

The most significant cluster of A549 (cluster 5) contained enrichment of genes located in nine of the 18 previously identified chromosomal regions with copy number changes in asbestos-associated lung cancer [8], i.e., 11q13, 19p13, 9q34, 16p13, 1p36, 17p13, 5q35, 3p21, and 22q13 (p < 0.01). Also cluster 2 of A549 contained enrichment of genes in 5 regions corresponding to the asbestos-associated regions in lung cancer. In total, clusters 5 and 2 contained enrichment of genes in 23 and 9 regions, respectively. The less significant clusters 3, 7, and 13 in Beas-2B contained enrichment of genes in 3 to 5 regions and cluster 2 in MeT5A contained enrichment of genes in 5 regions corresponding to the asbestos-associated regions. Overall, these clusters 3, 7 and 13 in Beas-2B and 2 in MeT5A contained enrichment of genes in 8, 14, 7, and 11 regions (p < 0.01), respectively (data obtained upon request).

Based on the literature, we examined 559 previously identified asbestos-associated genes to check their representation in the clusters. The Affymetrix probe sets corresponding to the listed genes were obtained with the data mining tool BioMart at Ensembl [12], based on the HGNC symbols. A total of 1,362 probe sets in the array represented the 559 asbestos-associated genes. Table 3 lists 55 of the probe sets corresponding to the previously known potential asbestos-, ROS- or mesothelioma-associated genes that were included in significant clusters in all cell lines. It is noteworthy that five collagen genes were recognized in significant clusters in all cell lines. Changes in the expression of procollagen is known to accompany the altered expression of *TGF-β *and *FN1 *following amosite asbestos exposure [13].

### Canonical Correlation Analysis Results

Canonical correlation analysis (CCA) was used to identify genes that contribute to the statistical dependencies between the A549 and Beas-2B cell lines. The MeT5A cell line was excluded due to the scarce time points. Measurements of differential gene expression between exposed and non-exposed cells were used for both cell lines in the analysis. Genes whose activation patterns show dependencies between two asbestos-exposed cell lines are more likely to be related to the asbestos exposure.

To achieve robust results, interpretation of the results was focused on gene groups rather than individual genes. We hypothesized that asbestos effects are spatially localized in chromosomes, and tested whether certain chromosomal regions were enriched in the gene list obtained by CCA. These regions could be potentially asbestos-affected (asbestos hotspots), and common to the cell lines.

Table 4 lists the most significant 21 regions (p-value < 0.03; q-value < 0.38). The number of genes contributing to the significance ranged from 1 to 71 genes (Additional file 5: CCA).

In the cluster analysis, the most significant cluster in Beas-2B (cluster 8; enrichment of genes in 10 regions) contained 3 regions corresponding to the regions identified in CCA (Table 4) and cluster 1 in MeT5A (enrichment of genes in 8 regions) contained 4 regions matching the CCA results, whereas none of the clusters in A549 had more than one region corresponding to the CCA results. Both the cluster analysis and CCA aim at revealing asbestos-related response, but they focus on different aspects of the data. In the three MeT5A clusters (enrichment of genes in 29 regions in total), we observed enrichment of genes in 7 regions corresponding to the CCA results, emphasizing that the changes do seem to reflect asbestos damage also in mesothelial cells.

Biological processes, such as "positive regulation of transcription, DNA-dependent" and "negative regulation of survival gene product activity", seen in the cluster and GO analyses (Table 2), were also associated with the genes in the regions of the CCA. Other processes that were found both in the CCA and the GO analysis were "calcium ion homeostasis", "frizzled signaling pathway", and "frizzled-2 signaling pathway", all known to contain asbestos associated genes [14]. These analyses are not, however, directly comparable, since they concentrate on different aspects of the data.

The CCA results were also correlated with the recently described germline 8-oxoguanine-rich regions in the human genome and the most significant 10 regions were found to be significantly associated with the 8oxoG regions (p = 0.024). These 8oxoG regions correlate with chromosomal regions that are frequently involved in recombination in the human genome. The regions affected by recombination are claimed to be more prone to damage through strand breaks [15].

## Discussion

We exposed transformed lung epithelial and mesothelial cells to asbestos and hybridized the samples from 3 to 6 time points to Affymetrix oligonucleotide arrays. The expression results were analyzed using three different methods to profile the expression pattern evoked by asbestos exposure (Fig. 1). The aim of this study was to profile gene expression at a genome-wide level to enable also other researchers to choose possible candidates for further investigation. Therefore we do not intend to discuss all the new potentially asbestos-related genes, but rather to select some examples. We are well aware that single genes are of little interest in profiling experiments when thousands of genes are evaluated. The genes we discuss here are some of those highlighted in three very diverse analyses. For complete results, see Additional files 1, 2, 3, 4, 5: GOanalysis, Cluster_analysis, CCA, A549clusters and Beas2Bclusters.

Many of the measured expression changes are probably due to the triggering of various universal cellular responses to foreign toxic substances, e.g., apoptosis or cell death. We anticipated that the use of three different cell lines would help us to pinpoint the specific asbestos-related effects. I.e. by comparing and identifying changes common to all cell lines, we expected to be able to neglect the expression changes associated with the malignancy or cell type and to concentrate on specific asbestos-related changes common to all cell types. Indeed, the number of GO terms shared by the cell lines increased with time, implying that response to the exposure occurs through the same pathways. Furthermore, the number of downregulated biological processes in each cell line increased with time, while the number of upregulated processes decreased (Fig. 1, bottom charts). This could be a consequence of apoptosis or cell death related functions. However, the failure of apoptotic functions to trigger cell death has been proposed to precede carcinogenic changes in a cell [16,17], making also the pathways and genes involved in the apoptotic processes worth investigating.

The asbestos associated genes *NFKB2 *and *IKBKB *[18,19] were present in the significant clusters of all cell lines (Table 3) and the GO term, "positive regulation of I-kappaB kinase/NF-kappaB cascade", associated with these genes was common for all the cell lines in both the cluster analysis and the GO analysis (Table 2). In addition, the genes within the GO term "cytoplasmic sequestering of NF-kappa B" were downregulated after 48h of asbestos exposure in all cell lines (Table 1). *NFKB *is known to be upregulated by asbestos and indeed, both the pathways and the probe sets corresponding to this gene exhibited upregulation at the initial time points, but downregulation at the later time points. This might be due to the fact that cells treated with asbestos often undergo apoptosis or cell death, whereas *NFKB *activation is involved in inhibition of apoptosis and cell survival [7].

Thioredoxin (*TXN*) and thioredoxin reductase (*TXNRD1*) downregulate apoptotic processes. These genes have been found to be upregulated in malignant pleural mesothelioma [20,21] and TXN is also known to be involved in the DNA-binding activity of NFKB [22,23]. *TXNDC *is located in 14q22, found in the CCA results. This thioredoxin-related gene was also represented in a highly significant cluster in all three cell lines. It is also one of the genes contributing to differential expression of the GO term "positive regulation of transcription, DNA-dependent", which was downregulated in all cell lines after 48h of asbestos exposure (Table 1). This suggests that the activation of thioredoxin and thioredoxin-related products, and subsequently *NFKB*, is evoked by other asbestos-activated products, e.g., TNF-α, derived from cell types other than the epithelial cells.

*BNIP3L *is a potential tumor suppressor gene, associated with hypoxia-induced epithelial injury [24]. Similar to *TXNDC*, *BNIP3L *was represented in all analyses, i.e., in the region 8p21 respresented in the CCA results, in the significantly downregulated GO term "negative regulation of survival gene product activity" (Tables 1 and 4), and it was present in the highly significant clusters 1, 8, and 2 (A549, Beas-2B, and MeT5A, respectively). *TXNDC *and *BNIP3L *were the only genes represented with such high significance in all analysis results and they can be readily envisaged as being associated with asbestos-related cellular damage based on their functions and their relationships with previously known asbestos-associated genes.

Asbestos-associated protein kinase c, delta (*PKC-δ*)and *p*-adducin (*ADD*) [4,25] were represented in significant clusters in all cell lines and *ADD3 *was represented in the most significant cluster of all cell lines (Fig 2A, D and 2G). *ADD1 *has also been found to be upregulated in asbestos-exposed lung cancer patients [10]. Furthermore, *PKC-δ *maps to a chromosomal area closely adjacent to 3p21, a region that has recently been found to be more frequently affected by loss of heterozygosity in asbestos-exposed than in non-exposed lung cancer patients [8,26]. Crocidolite asbestos is believed to modulate the intracellular calcium levels through activation of *PKC*, pointing to a possible involvement of calcium related pathways and genes [4]. We found both calcium-related genes and GO terms in the results of all analyses.*CAMK2D *was represented in high-ranking clusters of all cell lines and the potential asbestos hotspot regions identified by CCA harbored *CAMK1*. In addition, many other genes belonging to calcium-related biological processes were represented in the CCA data (Additional file 5: CCA). The GO analysis revealed that the biological process "calcium ion transport" had been affected in both Beas-2B and MeT5A cells (Additional file 1: GOanalysis).

The only upregulated biological process common to all three cell lines at the 48h time point in the GO analysis was "sensory perception of smell", which has not previously been proposed to be asbestos-associated. The majority of the genes involved in this process are G protein coupled olfactory receptors and taste receptors. G proteins have been suggested to be involved in the respiratory burst (release of ROS) caused by asbestos [27]. Furthermore, many G protein-associated biological processes, e.g., "G-protein signaling, coupled to IP3 second messenger" and "G-protein coupled receptor protein signaling pathway", were represented in our results (see Additional files 1 and 5: GOanalysis and CCA). This could indicate that the expression of G proteins is specifically altered by asbestos, possibly causing the release of ROS, which is known to contribute to carcinogenesis and progression to malignancy [28].

The most significant region according to the CCA was 2p22 (Table 4). It precedes a region (2p21-p16.3) that we have previously shown to be differentially altered in lung carcinomas of asbestos-exposed patients than in non-exposed patients' carcinomas [8]. A region homologous to the human 2p25-p21 has been reported to be amplified in radon-induced rat lung tumors [29], which could point to preferential damage that, for example, ROS production caused in this region of the genome. Additionally, a *c-fos*-like gene (*FOSL2*), which maps close to 2p21-p16.3, was present in significant clusters of all cell lines (Table 3). The early response genes *c-fos *and *c-jun *are closely linked to asbestos exposure [30]. Our results also revealed that the GO term "negative regulation of JNK activity", which regulates JUN kinase activity, was downregulated at the 48h time point in all cell lines (Table 1). Based on these results, the short arm of chromosome 2 could be an important potential target for DNA damage by asbestos, either directly or indirectly through ROS or other by-products. It is noteworthy that the probe sets located in 2p were substantially enriched in the highly significant clusters 9 of A549 (p = 0.00032) and 5 of Beas-2B (p = 0.000114) (data not shown).

Some of the crucial chromosomal aberrations in mesothelioma occur in 3, 4q, 5p, 6q, 8q22-q23, 9p, 14q12-q24 and 15q [9,31-35]. These regions correspond to the CCA results, strengthening the hypothesis that some chromosomal regions could be more prone to DNA damage caused by asbestos. Additionally, CCA revealed two interesting integrin genes in 2q31, *ITGA4 *and *ITGA6*. *ITGA4 *was significantly underexpressed in asbestos-exposed lung cancer patients [10] and *ITGA6 *has been associated with asbestos [6,36]. The long arm of chromosome 2 contains two fragile sites, FRA2G and FRA2H closely preceding and adjacent, respectively, to the region in the CCA results, suggesting that damage in these areas could cause specific changes in the expression of genes localized between the fragile sites. We have previously found fragile sites to be associated with asbestos-related copy number aberrations in lung cancer patients [8]. Furthermore, the *ITGA *genes are involved in calcium ion binding functions.

The significant clusters of the differentially expressed genes in the A549 cell line contained more genes located in regions corresponding to regions with differential copy number changes in asbestos-related lung cancer than was the case with the Beas-2B and MeT5A cell lines. This could possibly be attributable to the fact that A549 is a malignancy-derived cell line from a lung adenocarcinoma, whereas Beas-2B and MeT5A are SV40-transformed non-malignant cells that may exhibit the early effects of asbestos more clearly. This hypothesis is strengthened by the finding of enrichment of differentially expressed genes in regions corresponding to the CCA results in the clusters of the MeT5A cells, although this cell line was not included in the CCA.

## Conclusion

This study describes the asbestos-related gene expression profiles in lung epithelial and mesothelial cells at different time points. Clearly, *in vitro *experiments can never precisely reflect the conditions and mechanisms *in vivo*; for example, interactions between inflammatory cells and with the extra-cellular matrix are missing. Furthermore, both Beas-2B and MeT5A are SV40-immortalized cells which may contribute to some of the gene expression changes. However, efficient use of controls and careful selection of bioinformatics methods should account for these limitations and minimize the false negatives and positives. We expect these expression profiles may provide a better understanding of the mechanisms behind asbestos-associated disease, especially when correlated with gene expression data and CGH array data from asbestos-exposed lung cancer and mesothelioma patients [6,8-10,36-39].

## Methods

### Cell Lines

Human lung adenocarcinoma (A549) cells, human SV40-transformed bronchial epithelial (Beas-2B) cells, and SV40-immortalized pleural mesothelial (MeT5A) cells (American Type Culture Collection, Rockville, MD, USA) are well characterized and have been widely used as cell lines in pulmonary research [21,40-42]. The cells were cultured as described previously [43-46]. Briefly, A549 cells (American Type Culture Collection, Rockville, MD) were cultured in nutrient mixture F-12 growth medium supplemented with 15% fetal calf serum (FBS), 100 U/ml penicillin and 100 mg/ml streptomycin at 37°C in a 5% CO_2 _atmosphere. Beas-2B cells (National Cancer Institute, Laboratory of Human Carcinogenesis) were cultured according to the manufacturer' instructions (bronchial epithelial cell growth medium [BEGM]; Clonetics Inc., San Diego, CA). MeT5A cells were cultured using RPMI 1640 medium supplemented with 10% heat inactivated FBS, 0.003% L-glutamine, 100U/ml penicillin, and 100 mg/ml streptomycin at 37°C in a 5 % CO_2 _atmosphere.

Semi confluent cell cultures were exposed to crocidolite (International Union Against Cancer, Johannesburg, South Africa) (2 βg/cm^2 ^for A549 and Beas-2B cells and 1 βg/cm^2 ^for MeT5A cells) for different time points in the culture medium. The fibre doses were based on previous studies in our laboratory and by others [44-46]. Samples were collected from each cell line before any asbestos exposure or treatment (0h), from asbestos-exposed and control (not exposed, parallel cultures) A549 and Beas-2B cells at 1h, 6h, 24h, and 48h, with additional 7 days for A549. Samples from the MeT5A cell line were collected at 1h and 48h from exposed and parallel non-exposed control cells. The cultures and exposures were conducted on three or more separate tissue culture plates (T25 and T75), and the cells were pooled before hybridization to eliminate the need of biological replicate hybridizations. RNA was extracted and purified using Qiagen RNeasy kit (Qiagen Inc., Valencia, CA, USA) and RNA quality was measured using Agilent's BioAnalyzer (Agilent Technologies, Palo Alto, CA).

### Gene Expression Microarray

All samples, including one replicate from any time point for each cell line, were hybridized to Affymetrix Human Genome U133 Plus 2.0 oligonucleotide microarrays (Affymetrix, Santa Clara, CA). Reverse transcription of 5 βg high-quality total RNA to cDNA was carried out using the Superscript Double Stranded cDNA Synthesis kit (Invitrogen, Paisley, UK). The cDNA was linearly amplified and *in vitro *transcription reactions using the BioArray high-yield RNA transcript labelling kit (T7; Enzo Life Sciences, Farmingdale, NY) were carried out to produce biotinylated CTP and UTP-labelled cRNA. Labelled and fragmented cRNA was then hybridized to the Affymetrix microarrays for 16 h at 45°C in a rotating oven (60 rpm). The arrays were washed and stained with streptavidin-phycoerythrin (SAPE) in a Fluidics station 450 [47], and scanned with Affymetrix GeneChip Scanner 3000. The image was analyzed using the GeneChip operating software (GCOS; Affymetrix, Sacramento, CA) and comparison analysis was done according to the instructions provided by the manufacturer.

### Gene Expression Data Analysis

Affymetrix Analysis Suite v. 5 (MAS-5) was used to scale the arrays to the target value of 100 and to define the absent and present calls. Samples with a background of 35–70 and housekeeping control genes signal ratios close to 1.0 were included in the data analysis.

The hybridization data was pre-processed using RMA (Robust Multi-array Average) [48] with default settings (i.e., quantile normalization) in R. AFFX control sets and probe sets lacking GeneID information were excluded from the analysis. RMA pre-processing, designed to enhance the comparability of expression values between separate arrays, produces a single logarithmic expression value for each probe set in the Affymetrix arrays. When two arrays were used to measure the same experimental conditions (replicates), the mean of the two RMA values was used as the expression value. The technical replicates from each cell line correlated within acceptable values. Because Affymetrix oligonucleotide arrays are considered reliable, we did not use additional replicates.

Gene and chromosome band assignments were obtained from the BioConductor package 'hgu133plus2', version 1.10.0 [49]. Only unique assignments to properly named chromosome bands were used in the analysis.

### Bioinformatics Analysis

#### Gene Ontology Analysis

A statistical analysis of Gene Ontology (GO) annotation terms similar to that described by Breitling et al. [50] was performed to identify asbestos exposure-associated biological processes.

The analysis was carried out at each time point for both under- and overexpressed groups of genes. All cell lines were analyzed separately. Briefly, genes were first rank-ordered according to their logarithmic fold-change values between exposed cases and controls. When multiple probe sets corresponded to a unique gene (GeneID), we chose the '_at' set with the highest overall expression level in the three cell lines. When no '_at' sets were available, one of the sets was chosen at random. Genes involved in each biological process were assigned and a hypergeometric distribution was used for the statistical evaluation of enriched terms. Affected biological processes were determined by using the iGA algorithm of Breitling et al [50]. For a given gene class, iGA computes the minimal class-wise hypergeometric "p-value". The significance of this statistical indicator was here assessed by comparing its value against a distribution from 10 000 random permutations of the data. GO terms with permuted p-value of less than 0.01 were considered interesting.

To detect the most detailed (with the least genes) biological processes affected, the GO terms were ordered in branches according to their parent-child relationships. The branches form a tree-like structure where processes in each branch are related. Due to this close relationship, we assumed that truly affected processes should be detected on several levels of the tree. We therefore identified branches with at least three affected terms (p < 0.01).

#### Cluster Analysis

After RMA pre-processing and averaging of replicates, a differential expression time series of each probe set was formed by subtracting the expression values measured in the non-exposed control from the values in the asbestos exposed sample at each time point.

Probe sets were removed before cluster analysis (i) when none of the time points showed an approximate >1.4-fold (v2) difference between the non-exposed and exposed cases, (ii) when the probe sets did not have an associated gene title, and (iii) when the "present/marginal/absent" expression rating system used in Affymetrix microarray analysis software [47] (here, an open source implementation [51] of the Affymetrix algorithms was used instead) declared a probe set as absent in all microarrays relevant to the experiment. Each probe set was treated as the sole representative of a gene. The number of probe sets remaining after the pruning procedures was 7 538 (MeT5A), 12 436 (A549), or 16 640 (Beas-2B). A total of 19 710 out of the 54 675 different probe sets on the Affymetrix array were included in the cluster analysis of at least one cell line.

The reduced data set from each cell line was clustered using an algorithm specifically designed for short time series expression data [52,53]. Significant clusters were not grouped as in the original paper. The clustering procedure assigned each probe set into a single cluster. The total number of clusters was set to 50, and the algorithm labelled some as being statistically significant (p < 0.05, Bonferroni corrected) using a permutation test.

Furthermore, the enriched biological processes (GO) as well as enriched chromosomal regions (both referred to as "terms" in the following) for each cluster were accounted for by calculating the probability of having at least the observed number of probe sets associated with a given term, assuming a random selection of probe sets. All 54 675 probe sets on the microarray were used as a reference set. The possible enrichment of the terms in different subsets (clusters) of the reference set was evaluated by computing p-values from the hypergeometric distribution.

#### Canonical Correlation Analysis

CCA [54] was performed on the A549 and Beas-2B cell lines. This method describes the shared variation between two data sets. The MeT5A cell line was omitted from the analysis due to the scarcity of time points. Multiple probe sets corresponding to the same gene were treated as in the GO analysis. Genes were ordered, based on the results from CCA, according to their contribution to the dependencies of the two data sets. This was measured by the squared sum of CCA projection scores. Enrichment in 307 chromosome bands was tested. The p-values were evaluated based on permutation test as in the GO analysis.

## Authors' contributions

PN and PL carried out the microarray hybridizations, participated in the coordination of the bioinformatics analysis design, interpreted the analysis results and drafted the manuscript. MK performed the pre-processing of the data and the cluster analysis. LL performed the CCA. SR performed the GO analysis. JH and SKa coordinated and participated in the choice and design of the bioinformatics analyses. VK arranged the cell cultures and exposures. SA, VK and SKn conceived the study and participated in its design, as well as coordinated the drafting of the manuscript. All authors read and approved the final manuscript.

## Supplementary Material

Additional File 1 GO analysis. The file contains enriched biological processes in each time point and each cell line. Click here for file

Additional File 2 A549 clusters. The file contains profiles from all significant clusters in cell line A549. The X-axis shows the time points from 0h-7 days) and the Y-axis shows an expression ratio profile that is representative of all probe sets in the cluster. Due to the correlation based distance measure used in the clustering method (Ernst et al, 2005), the scale of the Y-axis is not significant. Click here for file

Additional File 3 Beas-2B clusters. The file contains profiles from all significant clusters in cell line Beas-2B. The X-axis shows the time points from 0h-48h and the Y-axis shows an expression ratio profile that is representative of all probe sets in the cluster. Due to the correlation based distance measure used in the clustering method (Ernst et al, 2005), the scale of the Y-axis is not significant. Click here for file

Additional File 4 Cluster analysis. The file contains lists of all genes included in all significant clusters of each cell line. Click here for file

Additional File 5 Canonical correlation analysis. The file contains list of genes contributing to the enrichment of the chromosomal regions that contribute to the common correlation between the cell lines A549 and Beas-2B. Click here for file

## Figures and Tables

**Table 1:** GO analysis results. Differentially expressed biological processes common in all cell lines after 48 h of exposure.

GO term*	**P-value/expression**	**Genes contributing to the differential expression**	**Total no. of genes in the GO term**
Negative regulation of helicase activity	< 0.01/down	*TP53*	2
Regulation of mitochondrial membrane permeability	< 0.01/down	*TP53*	8
Positive regulation of transcription, DNA-dependent	< 0.005/down	*ARID1A, THRAP3, GLIS1, FOXD3, ARHGEF11, ATF6, ESRRG, NCOA1, FHL2, CTNNB1, SCAP, SMARCC1, ZBTB38, TP73L, PPARGC1A, NARG1, MAML3, IL31RA, CDK7, RNF14, PPARGC1B, NSD1, MAML1, SQSTM1, NOTCH4, HMGA1, TBN /// LOC441149, TBN, TRERF1, FOXO3A, ASF1A, PLAGL1, ARID1B, CREB5, SMARCD3, GATA4, GLIS3, RXRA, NCOA4, EHF, HTATIP, RBM14, RBM14 /// MGC15912, HBXAP, CRSP6, MAML2, ATF7IP, SURB7, SP1, SMARCC2, GRIP1, TBX5, RAN /// LOC391717, NARG1L, RB1, TXNDC, MED6, TP53BP1, ELL3, PIAS1, MKL2, TGFB1I1, CTCF, MAP2K3, PPARB, BRCA1, THRAP, PIAS2, SMAD2, ILF3, MECT1, CCNE1, MLL4, DYRK1B, NCOA3, NRIP1, RUNX1, SEC14L2, ATF4, PHF5A, G22P1, CRSP2, MED12*	848
Negative regulation of JNK activity	< 0.01/down	*HIPK3*	5
Prenylated protein catabolism	< 0.01/down	*PCYOX1*	4
Negative regulation of survival gene product activity	< 0.01/down	*BNIP3L, HRK, BCL2L1, BAX*	19
Ras protein signal transduction	< 0.01/down	*PARK7, MFN2, CNKSR1, LCK, NOTCH2, IQGAP3, SOS1, LZTFL1, RASSF1, MRAS, PLD1, G3BP2, FGF2, IQGAP2, KIAA1961, RAPGEF6, G3BP, RREB1, RGL2, PLCE1, SHOC2, RASGRP2, IGF1, RASGRP1, TPM1, IQGAP1, LAT, GAS8, GRAP, KSR, NF1, GRB2, RASGRP4, RRAS, NU62, NUP62 /// IL4I1, CRKL, GRAP2*	477
Cytoplasmic sequestering of NF-kappaB	< 0.005/down	*FAF1, IL10, G3BP2, NFKBIA, BCL3*	22
Regulation of MAPK activity	< 0.005/down	*MAP3K6, SHC1, RGS4, LAX1, KIAA1804, TRIB2, SPRED2, DUSP2, MAP3K2, ZAK, PROK2, PIK3CB, ADRA2, EGF, FGF2, PRKAA1, PPP2, DUSP22, DAXX, MAPK3K5, DBNL, PP2CB, TRIB1, RGS3, PLCE1, DUSP8, HPK3, MAP3K11, PP2R1B, DUSP6, MBIP, MAP4K5, MAP3K9, SPRED1, HSPC121, GPS2, ADORA2B, GPS1, EDG5, PKN1, MAP4K1, MAP3K10, C5R1, PPP2R1, TRIB3, BIRC7, DUSP9*	244
Sensory perception of smell	< 0.005/up	*TAS1R1, TAS1R2, OR10K1, OR10, OR2C3, OR1C1, OR2W3, OR2L13, OR2M4, ADCY3, GRM7, SLIT2, YGT2A1, TAS2R1, OR2B2, ORB2B6, OR2W1, OR2B3, OR5U1, OR5V1, OR12D3, OR5V1 /// OR12D3, OR12D2, OR11A1, OR10C1, OR2H1, OR2H2, CYB5R4, TAS2R16, GRM8, TAS2R3, TAS2R4, TAS2R5, TAS2R38, OR6W1, TAS2R39, TAS2R40, TAS2R41, OR6B1, OR2S2, OR1J2, OR1J4, OR1Q1, OR51E1, OR51E2, OR52A1, OR51B4, OR51B2, OR51M1, OR51I1, OR51I2, OR52D1, OR6A2, OR10A4, OR5P2, OR5P3, PR5E1, OR10A3, OR5I1, OMP, OR8G1 /// LOC442754, OR8D1, OR8B2, OR8B8, TAS2R45, TAS2R7, TAS2R8, TAS2R9, TAS2R1, TAS2R13, TAS2R14, TAS2R50, OR4N4, OR1F1, OR1F2, OR2C1, OR1D2, OR1G1, OR1A2, OR1A1, OR3A2, OR3A1, OR1E1 /// OR1E2, OR4D1, OR4D2, OR7D2, OR7E19, OR7C1, OR7A5, OR7A10, OR7A17, OR7C2, OR1I1, OR10H2, C5R1*	2589

*see Additional file 1: GOanalysis for all significant GO terms at each time point.

**Table 2:** Cluster analysis versus GO analysis. Common up- and downregulated biological processes (GO) in the cluster analysis and the GO analysis. Processes significant in more than one time point of any cell line in the GO analysis and occurring in more than one cluster of any cell line.

**GO biological process**	**Cluster analysis**			**GO analysis**
		
	**A549 cluster number (uncorrected p-value)**	**Beas-2B cluster number (uncorrected p-value)**	**MeT5A cluster number (uncorrected p-value)**	
Antigen processing, endogenous antigen via MHC class I	5 (9.52 × 10^-10^)	12 (1.31 × 10^-3^)		A549 1 h up A549 24 h down
Apoptotic program	9 (2.58 × 10^-3^)	6 (3.00 × 10^-3^)	2 (2.55 × 10^-4^)	Beas-2B 24 h down A549 48 h down
Cytoskeletal anchoring	9 (2.04 × 10^-3^)	6 (2.44 × 10^-3^), 16 (1.38 × 10^-3^)	2 (2.85 × 10^-3^)	A549 24 h down MeT5A 48 h down
Nucleosome assembly	1 (5.68 × 10^-4^)	3 (2.52 × 10^-3^), 5 (4.02 × 10^-3^), 13 (1.13 × 10^-3^)		Beas-2B 1 h down MeT5A 1 h up
Regulation of DNA replication	5 (9.56 × 10^-3^)	11 (3.02 × 10^-3^)		A549 1 h up A549 6 h up Beas-2B 24 h down Beas-2B 48 h down
Regulation of translational initiation	1 (7.00 × 10^-5^)		2 (5.73 × 10^-3^)	A549 48 h down Beas-2B 6 h down Beas-2B 48 h down MeT5A 1 h up
Positive regulation of transcription, DNA-dependent	8 (1.59 × 10^-3^)		2 (3.73 × 10^-3^)	A549 48h down Beas-2B 6h down MeT5A 1h up MeT5A 48h down
Positive regulation of I-kappaB kinase/NF-kappaB cascade	1 (5.98 × 10^-3^), 4 (1.03 × 10^-4^)	1 (2.36 × 10^-3^), 16 (1.17 × 10^-4^)	2 (2.07 × 10^-3^)	A549 6h up A549 24h down A549 48h down Beas-2B 6h down Beas-2B 24h down Beas-2B 48h down MeT5A 1h up
Negative regulation of survival gene product activity	8 (9.92 × 10^-3^)		2 (4.06 × 10^-4^)	A549 6h up A549 24h down A549 48h down Beas-2B 1h down Beas-2B 24h down Beas-2B 48h down MeT5A 1h up MeT5A 48h down

**Table 3:** Asbestos-related genes. Previously identified potential asbestos-associated genes represented in significant clusters of all cell lines. (Affymetrix IDs are available upon request)

**Asbestos-associated gene**	**A549 cluster**	**Beas-2B cluster**	**MeT5A cluster**	**Chr. loc**
Mesothelioma-associated genes*				
*BIRC5*	5	13	2	17q25
*BTG2*	5	7	3	1q32
*CALU*	9	8	3	7q32
*CALU*	9	8	3	7q32
*COL1A1*	5	10	2	17q21.33
*COL4A1*	5	8	2	13q34
*COL5A1*	5	14	2	9q34
*COL6A2*	5	3	2	21q22
*FN1*	9	6	3	2q34
*HMGB1*	9	3	2	13q12
*HNRPH1*	5	7	2	5q35
*IGFBP4*	4	10	3	17q21.2
*KIBRA*	5	6	3	5q34
*LU*	2	3	2	19q13
*LU*	2	10	3	19q13
*NF2*	5	7	2	22q12
*PDLIM7*	2	7	2	5q35
*PDLIM7*	5	7	2	5q35
*PEG10*	9	8	3	7q21
*POM121*	4	13	2	7q11
*PTPRF*	5	13	2	1p34
*PXN*	5	13	2	12q24
*TPD52L1*	5	6	1	6q22.31
*TP53*	5	7	2	17p13
*TXN*	11	1	1	9q31
*VEGF*	9	8	3	6p12
*VEGF*	9	8	3	6p12
*VIL2*	8	16	2	6q25.3
Human homologs of asbestos associated murine genes^†^				
*BIRC4*	6	7	1	Xq25
*BIRC4*	7	13	1	Xq25
*BMP2*	5	14	1	20p12
*CD44*	9	14	2	11p13
*CSNK2A1*	9	13	2	20p13
*CTSB*	1	14	1	8p22
*FAS*	1	8	1	10q24
*FAS*	1	8	1	10q24
*IQGAP1*	9	6	3	15q26
*MAP4K4*	1	8	3	2q11.2
*PLEC1*	9	6	2	8q24
*PTPN14*	9	6	3	1q32
*PTPRF*	5	13	2	1p34
*RHOA*	9	6	2	3p21
*TP53*	5	7	2	17p13
*TRAM1*	5	8	2	8q13
*VAV2*	5	13	2	9q34
Other asbestos-associated genes^‡^				
*ADD3*	5	8	3	10q24
*CASP8*	9	6	2	2q33.1
*CDK10*	5	13	2	16q24
*COL12A1*	9	8	2	6q13-14.1
*FOSL2*	2	3	3	2p23
*IKBKB*	9	16	2	8p11
*KRAS*	7	13	1	12p12
*L1CAM*	2	11	2	Xq28
*MAPK1*	3	5	3	22q11
*MAPK1*	9	6	2	22q11
*MAPK1*	9	6	2	22q11
*NFKB2*	5	13	3	10q24
*PCDHGA6*^§^	12	10	3	5q31
*PRKCD*	5	6	3	3p21

* [17, 36–39]

^†^[6, 20]

^‡ ^[4, 18, 19, 55, 56]

^§ ^The probe also corresponds to PCDHGC3 /// PCDHGB4 /// PCDHGA8 /// PCDHGA12 /// PCDHGC5 /// PCDHGC4 /// PCDHGB7 /// PCDHGB6 /// PCDHGB5 /// PCDHGB3 /// PCDHGB2 /// PCDHGB1 /// PCDHGA11 /// PCDHGA10 /// PCDHGA9 /// PCDHGA7 /// PCDHGA6 /// PCDHGA5 /// PCDHGA4 /// PCDHGA3 /// PCDHGA2 /// PCDHGA1

**Table 4:** Chromosome bands from the CCA results. Potential asbestos hotspots, some of which contain known asbestos-related genes. Expected false discovery rate of < 38%.

Region	P-value/q-value	No. of genes in the region with the most significant contribution to the common correlation*	Examples of genes
2p22	0.00260/0.3367	10	*MAP4K3*^†^*, CEBPZ*^§^,*QPCT*^§^
9p13	0.00700/0.3367	71	*FANCG*^†^*, IGFBPL1*^†^*, CCL19*^†^*MELK*^†^
14q21	0.00720/0.3367	22	*FANCM*^†^*, CDKL1*^†^
5p13	0.00880/0.3367	5	
9q21	0.00970/0.3367	29	*PCSK5*^†^
5q13	0.01010/0.3367	9	*F2RL1*
8q22	0.01160/0.3367	59	*CTHRC1*^†^*, TP53INP1*^†^*, KLF10*^†^
2q36	0.01260/0.3367	29	*MRPL44*^†^
6q13	0.01300/0.3367	9	
2q31	0.01370/0.3367	28	*KIAA1715, AGPS, ITGA4*^†^*, ITGA6*^†^
3q29	0.01410/0.3367	54	*DLG1*
3p25	0.01480/0.3367	67	*COLQ*^†^*, CAMK1*^†^*, RAF1*^†^*, TMEM43*,*LOC51244*
15q12	0.0176/0.3471	2	
4q11	0.0178/0.3471	6	
8p21	0.0196/0.35672	70	*BNIP3L, GSR*^§^
12q12	0.0225/0.3705	7	
14q22	0.0258/0.3705	29	*TXNDC*
2q34	0.0266/0.3705	1	
15q13	0.0272/0.3705	9	
5q15	0.0279/0.3705	2	*KIAA0372*^§^
3q23	0.0285/0.3705	4	

* see Additional file 5: CCA for complete lists of the genes

^† ^Known or related to a known asbestos-associated gene. *ITGA4 *also differentially expressed in asbestos-exposed lung cancer patients as compared to non-exposed lung cancer patients [10].

^§ ^The gene also shows differential copy number in CGH array data of asbestos-exposed lung cancer patients, (data not shown, available at request)
